# Endemics and the Soil

**Published:** 1871-05

**Authors:** 


Endemics and the Soil.—At the last meeting of the British Association in Liverpool, Dr. Moffat, of Hawarden, read an interesting paper on "Geological Systems and Endemic Diseases," showing that the soil had an influence on the composition of the cereal plants grown upon it, and on the diseases to which the inhabitants are subject. The district in which he practices consists geologically of the carboniferous and new red sandstone, or Cheshire sandstone systems.

Anæmia with goitre is prevalent among those living on the carboniferous systems, whilst it is almost unknown among those living on the new red sandstone system; and consumption is also more prevalent amongst the inhabitants of the former. Dr. Moffat has found by analysis that the wheat grown on the soil of the Cheshire sandstone, contains the largest quantity of ash, and that there is a larger quantity of phosphoric acid grown on the soils of the carboniferous and mill-stone grit systems. He has calculated that each inhabitant of the Cheshire sandstone, if he consumes a pound of wheat daily, takes in nearly five grains per day of the sesquioxide of iron more than the inhabitant of the carboniferous system, who seems, therefore, to be subject to anæmia in consequence of deficiency of iron and phosphoric acid in his food.

It is not only in the wheat grown upon the carboniferous system that there is a deficiency in the quantity of iron and the phosphates, according to Dr. Moffat, but also in the blood of the animals reared upon it. He stated that sheep were liable to anæmia, which he attributed to sheep-walks being upon trap and limestone hills, in the soil of which there is but little, if any, iron.—*National Medical Journal.*

